# Clinical use of biomarkers in the era of Alzheimer's disease treatments

**DOI:** 10.1002/alz.14201

**Published:** 2024-12-30

**Authors:** Lawren VandeVrede, Suzanne E. Schindler

**Affiliations:** ^1^ Department of Neurology University of California San Francisco San Francisco California USA; ^2^ Department of Neurology and the Knight Alzheimer Disease Research Center Washington University School of Medicine St. Louis Missouri USA

**Keywords:** Alzheimer's disease, amyloid, biomarkers, blood, cerebrospinal fluid, dementia, diagnosis, disclosure, positron emission tomography, treatment

## Abstract

With the advent of treatments that specifically target Alzheimer's disease brain pathology, biomarker tests will become an increasingly important part of the routine clinical evaluation of cognitive impairment and guide clinical decision making. Clinicians must ensure they are using accurate and well‐validated biomarker tests and select the most appropriate testing modality for each patient based on individual and practical considerations. The interpretation of test results may be complex and depends on the pre‐test probability and test‐specific factors. Biomarker results must be presented and discussed with patients in a process that is sensitive to the major implications of the results and is carefully connected to diagnosis, prognosis, and management. Advances in treatments for Alzheimer's disease will likely require non‐dementia specialists to use biomarkers, necessitating major educational efforts. In the new era of Alzheimer's disease treatments, biomarkers are essential tools that will be integrated into all aspects of dementia diagnosis and care.

## 1 A HISTORICAL PERSPECTIVE

In 1906, Alois Alzheimer described amyloid plaques and neurofibrillary tangles in the brain of Auguste Deter, a woman who died from a progressive dementia syndrome.^1^ For the next 100 years, the disease that came to bear his name, Alzheimer's disease (AD), could only be diagnosed definitively after an autopsy demonstrated the defining neuropathological hallmarks of AD: amyloid plaques and neurofibrillary tangles. Clinicians learned that certain clinical features predicted the presence of AD pathology at autopsy, including an insidious onset of cognitive impairment, early amnestic features, and a slow progression of cognitive decline over years.^2^ Additionally, several atypical clinical syndromes, characterized by prominent changes in behavior, vision, or language, were found to be associated with AD brain pathology. While careful clinical evaluation improved diagnostic certainty, a significant percentage of individuals with clinical syndromes typical of AD dementia lacked AD pathology at autopsy, even when evaluated at expert centers.^3^ Additionally, the misdiagnosis of AD dementia severely hindered early clinical trials aiming to target the neuropathology specific to AD; a significant percentage of participants enrolled in early AD clinical trials based on clinical criteria alone were later found to lack AD pathology.

- Biomarkers will be integrated into all aspects of dementia diagnosis and care.
- The optimal biomarker test depends on patient‐related and practical considerations.
- Test interpretation depends on the pre‐test probability and test‐specific factors.
- Biomarker results must be carefully presented and discussed with patients.

Fortunately, biomarker tests were developed that enabled detection of AD pathology in living individuals. In the late 1990s, it was found that lower levels of amyloid‐β peptide 42 (Aβ42) and higher levels of tau in the cerebrospinal fluid (CSF) were associated with AD dementia.^4^, ^5^ In 2004, the first radiotracer was described that bound to amyloid plaques and enabled imaging of brain amyloid burden via positron emission tomography (PET).^6^ In 2017 and 2018, the first of many AD blood tests demonstrated good performance in identifying individuals with amyloid pathology.^7^, ^8^ With the advent of AD biomarkers, clinical trials were more rigorously designed and therapeutics targeting AD pathology began to show efficacy in slowing cognitive decline.^9^, ^10^ AD biomarkers have now become essential tools in clinical trials to identify individuals with AD pathology and monitor the effects of treatments.^11^, ^12^

While multiple cerebrospinal fluid (CSF) biomarker tests and amyloid PET scans have been used in research studies and clinical trials for nearly two decades and have been clinically available for over a decade, they have been used infrequently in clinical practice until very recently. The perceived invasiveness, need for specialized providers and/or equipment, and expense of these biomarker modalities has set a relatively high threshold for performing clinical AD biomarker testing.^13^ However, the major reason for the low rate of biomarker testing was that patients and providers expected that biomarker testing would not significantly affect management or outcomes for most patients. Now that treatments are available that specifically target AD pathology, there is a greatly increased need for clinicians to not only accurately diagnose a patient's clinical syndrome, but also to use biomarkers to determine whether AD is the likely neuropathological etiology causing cognitive impairment.

## 2 EVALUATION OF PATIENTS WITH COGNITIVE IMPAIRMENT

As we enter the modern era of disease‐modifying therapeutics for AD, clinicians are expected to rapidly become familiar with a range of biomarker tests that can refine a differential diagnosis. While appropriate use criteria can provide guidance,^14^, ^15^, ^16^ deciding if and when to order testing must be informed by a discussion with the patient and their loved ones regarding the benefits and drawbacks of testing. Additionally, decisions about which test to order must balance the technical and diagnostic performance of specific tests with practical issues such as cost and availability and the preferences of the patient. Therefore, it remains of the utmost importance to first perform a comprehensive clinical evaluation of patients who present for evaluation of memory and thinking concerns, which then informs decisions about if and when biomarkers should be ordered, and which modality is most appropriate. Biomarkers are ancillary tools, not a substitute for clinical evaluation and informed clinical reasoning.

A thorough evaluation of cognitive impairment typically includes a detailed history, examination, cognitive testing, routine blood work, and structural brain imaging. Because AD pathology silently accumulates for many years before causing cognitive impairment, many older individuals have positive biomarker tests that reflect early AD pathology that may not yet be causing cognitive impairment.^17^ These individuals with preclinical AD may develop cognitive impairment for reasons unrelated to AD, but may be inaccurately diagnosed with AD dementia if only biomarker testing is considered. This is particularly true for patients diagnosed with mild cognitive impairment, which is common in older adults and may be due to potentially reversible etiologies such as medications, sleep disorders, medical issues, and mood disorders.^18^ Therefore, it remains essential to consider, evaluate, and treat non‐AD causes of cognitive impairment, including in patients with biomarker evidence of AD pathology.

The guiding principle in deciding whether to perform biomarker testing is that the result should be reasonably likely to provide a benefit to the patient. Now that specific treatments are available for early symptomatic AD, a common and compelling indication for biomarker testing is to determine whether patients who may be candidates for treatment are amyloid positive.^19^ If biomarker testing is being considered solely for the purpose of qualifying for AD‐specific treatments, the risks and benefits of both biomarker testing and potential therapeutic options should be discussed before ordering biomarker testing; after such a discussion, patients may decide they are not interested in treatments and therefore biomarker testing might not be indicated. However, biomarkers can be helpful to refine the clinical diagnosis even in the absence of treatment considerations. The diagnosis of AD is frequently affected by biomarker test results, especially when the diagnosis is uncertain or the clinical features are atypical.^20^ A more certain diagnosis may help clinicians provide more appropriate care and also help patients and their loves ones make more appropriate decisions, especially regarding future planning. Therefore, we advocate for clinicians to routinely offer AD biomarker testing to patients with cognitive impairment if AD is on the differential diagnosis.

For cognitively unimpaired patients and patients with subjective cognitive impairment, biomarker testing is not recommended as part of clinical care. There are currently no data demonstrating that biomarker testing positively affects the outcomes of cognitively unimpaired individuals. Clinical trials are underway to evaluate the efficacy of treatments to slow or prevent the onset of cognitive symptoms in cognitively unimpaired individuals with AD pathology and are highly promising,^21^, ^22^ but these trials have not yet been completed and it is uncertain whether they will show a positive result. While some cognitively unimpaired individuals might change their lifestyle if they obtained positive AD biomarker results, many of these lifestyle changes would be reasonable to implement in patients at risk for dementia regardless of biomarker results. Further, there may be negative consequences for cognitively unimpaired individuals of having positive AD biomarker results, such as losing eligibility for some forms of insurance or potentially being subject to discrimination.^23^ Therefore, until there is evidence that biomarker testing improves the outcomes of cognitively unimpaired patients, biomarker testing of these individuals outside of research studies or clinical trials likely has more risks than benefits.

## 3 BIOMARKER TESTS FOR AD PATHOLOGY

Provided that the available options reach a minimum level of accuracy, the best AD biomarker test to order is the one you can get. Practical issues frequently dictate medical decision making, and most clinics do not have all modalities of biomarker testing readily available. Setting up or scaling up some AD biomarker modalities, particularly CSF tests and PET, can be extremely challenging because of the need for specially trained personnel and/or expensive equipment.^24^ Even when biomarker testing is available, getting different insurances to cover testing often requires additional clinical time and support staff, especially if administrative hurdles and barriers exist that discourage costly testing. In the current era, multiple modalities are available for biomarker testing, and a natural experiment is taking place whereby different clinics are using different biomarker modalities based on their own local factors. The optimal biomarker modalities for uses like diagnosis, staging, prognosis, and/or treatment monitoring will become clearer over time, and multiple options might be available for each purpose.

### 3.1 Amyloid and tau PET

Amyloid and tau PET scans involve injection of a radiotracer that binds to amyloid plaques or neurofibrillary tangles, respectively, followed by imaging via PET. Patients are injected intravenously with a radioactive tracer and then lie on a cushioned table, which is moved into a donut‐shaped scanner. The PET scanner takes pictures of the brain and is much quieter than a magnetic resonance imaging (MRI) scan. The entire process, including the injection and scan, takes about 1 h. The radiation exposure is very low. Patients who are concerned about radiation exposure or who have had many x‐rays or imaging scans in the past or planned for the future should talk with their doctor about their risk for radiation‐related complications.

Several amyloid PET tracers are validated and FDA‐approved based on their agreement with autopsy‐confirmed AD neuropathology.^14^, ^25^ Most clinical amyloid PET scans are read visually by certified raters as either positive or negative, and no quantitative measure of amyloid burden is provided. In clinical practice, there is an approximately 14% disagreement between visual reads and rigorous quantitative measures of amyloid burden; much of this disagreement is for individuals with low levels of amyloid pathology.^26^ Tau PET scans are validated based on correlations with neurofibrillary tangles,^27^ and in clinical practice are read visually. Tau PET is strongly correlated with cognitive impairment and disease stage.^28^

The higher cost of PET has limited use of this modality in clinical care. Amyloid PET was not covered by insurance until very recently, making it unavailable to most patients outside of research or clinical trials. However, on October 12, 2023, Medicare revised its non‐coverage determination and started reimbursing for amyloid PET for the purpose of determining amyloid status. Other insurances still may not reimburse for amyloid PET, although increasingly it is reimbursed to determine amyloid status of patients who are candidates for AD‐specific treatments. Now that amyloid PET is covered by Medicare and some other insurances, both the availability and use of amyloid PET are increasing for diagnostic confirmation of amyloid pathology. Additionally, amyloid PET was used in clinical trials to demonstrate amyloid clearance,^9^, ^10^ so it may be useful in monitoring patients on amyloid‐lowering treatments. However, the frequency and number of amyloid PET scans required for monitoring amyloid burden, and whether insurance will cover the scans, remains uncertain. The role of tau PET in clinical practice is currently unclear because of high costs and very limited availability.

### 3.2 CSF tests

CSF is made by the brain, circulates around the brain, and reflects brain health. Changes in the concentrations of certain CSF proteins are strongly associated with AD pathology. CSF is collected via a lumbar puncture. A skilled clinician feels the patient's lower back, cleans it with a sterilizing solution, and injects a numbing medication. A very thin needle is then inserted into the low back in a specific location, and CSF slowly drips out. The procedure takes approximately 30 min. Lumbar puncture is very safe, but some patients have a sore back or headache after the procedure. In occasional cases (< 5%) when patients develop a headache due to a persistent leak of CSF, a second procedure can be performed to stop the headache by application of an epidural blood patch over the site of lumbar puncture. CSF collection may be more difficult in patients with scoliosis, prior lumbar back surgery, or severe lumbar adiposity, and CSF collection is relatively contraindicated in patients who are taking certain anti‐coagulant medications.

FDA‐approved CSF tests have been validated by demonstrating high agreement (approximately 90%) of CSF Aβ42/Aβ40, p‐tau181/Aβ42, or t‐tau/Aβ42 with visual read of amyloid PET scans. Disagreement between amyloid PET and CSF tests is most common in individuals with low levels of amyloid pathology. Also, CSF biomarkers change earlier in the course of AD compared to amyloid PET,^29^ so individuals with positive CSF biomarkers and negative amyloid PET may have very early AD pathology. The CSF measures that are currently used in the clinic are only weakly correlated with disease stage and provide no information on the regional distribution of AD pathology, although more informative measures are in development.^28^, ^30^, [Table alz14201-tbl-0001]

Some clinicians have used CSF tests in AD diagnosis for almost two decades. CSF testing has been the dominant AD biomarker modality used in the clinic because it was the only modality covered by insurance until recent coverage of amyloid PET by Medicare.^31^ CSF testing can also be used to evaluate for some non‐AD causes of cognitive impairment, including Creutzfeldt‐Jacob disease, autoimmune‐mediated encephalitis, and synucleinopathies, making this modality particularly helpful in patients with a wide differential diagnosis.^31^, ^32^ However, most patients have never undergone a lumbar puncture and some perceive it to be invasive and/or risky. Additionally, highly trained personnel are required to perform lumbar puncture and reimbursements typically do not fully cover the costs of performing the procedure.^24^ Overall, issues related to acceptability and accessibility have significantly limited the use of CSF tests in AD diagnosis.

### 3.3 Blood tests

Assays for some of the same analytes (e.g., Aβ42/Aβ40, p‐tau181, and p‐tau217) that were initially measured in CSF and found to be strongly associated with AD pathology have been translated into blood tests for AD.^33^, ^34^ Blood is collected via standard phlebotomy techniques. The procedure is very safe and well tolerated, making blood tests strongly preferred by patients over CSF tests.

No AD blood tests are yet FDA‐approved as in vitro diagnostics, but some are offered clinically as laboratory developed tests, which do not require rigorous clinical validation. Some tests have received FDA Breakthrough Status, but this is not equivalent to FDA approval and does not imply high accuracy. The accuracy of clinically available AD blood tests is widely variable, with some tests performing as well as FDA‐approved CSF tests in classification of amyloid status and other tests performing only slightly better than the flip of a coin.^35^, ^36^ Blood tests that include p‐tau217 generally have the highest associations with amyloid PET and tau PET measures.^37^, ^38^ Like CSF tests, blood tests do not provide information on the regional distribution of AD pathology, but it is possible that some plasma biomarkers might be useful in staging AD.^28^

Because of the high acceptability of blood tests and existing infrastructure for blood collection, blood tests are potentially rapidly deployable and highly scalable. Unlike PET or CSF tests, it is conceivable that the number of blood tests could increase by orders of magnitude over a few years. However, the variability in the accuracy and validation of AD blood tests represents a major obstacle to broader acceptance of these tests, as many clinicians are unsure about which specific tests are appropriate for clinical use. The costs of the tests are also variable and typically not reimbursed by insurance.

## 4 SELECTION OF AD BIOMARKER MODALITY

When selecting an AD biomarker test, the optimal modality for an individual patient may be affected by many different factors (Table 1). Patients who are concerned about radiation or who have severe claustrophobia may be unwilling or unable to undergo PET imaging. If a patient does not have insurance coverage, amyloid PET or CSF testing may be cost prohibitive, and blood tests may be preferred, especially if income‐dependent patient assistance programs reduce the cost of the blood tests. Lumbar puncture may be relatively contraindicated in patients taking certain anti‐coagulant medications. Some patients may be unwilling to undergo CSF testing due to perceptions about the invasiveness or risks of lumbar puncture. Additionally, CSF testing may be a poor option for some patients at risk for a difficult lumbar puncture due to scoliosis, prior lumbar back surgery, or severe lumbar adiposity. However, CSF testing may be the optimal biomarker modality for patients in whom the differential diagnosis includes certain non‐AD conditions that can be identified via specific CSF tests. Insurance‐related considerations may drive the choice of AD biomarker modality if the patient is a candidate for AD‐specific treatments, as some insurances will accept a positive amyloid PET or CSF test result but not a positive blood test result as adequate evidence of amyloid pathology. Notably, we have found that some insurances including Medicare may accept a positive blood test result as adequate evidence of amyloid pathology that enables initiation of AD‐specific treatments. If only lower accuracy or poorly validated AD blood tests are available, amyloid PET or CSF tests would be preferable given the major consequences of test results. Finally, certain comorbidities such as chronic kidney disease may cause false positive results for certain AD blood tests,^39^ so amyloid PET or CSF testing may be preferable in patients with significant medical comorbidities. However, some AD blood tests may perform relatively consistently despite the presence of medical comorbidities.^40^

**TABLE 1 alz14201-tbl-0001:** Patient‐specific factors that may affect the selection of biomarker modality.

Patient‐specific factors	Amyloid PET	CSF tests	Blood tests
Patient is very concerned about risks from radiation	↓	↑	↑
Patient has severe claustrophobia	↓	↑	↑
Patient lacks insurance coverage for biomarker testing and cost is a concern	↓	↓	↑
Patient is treated with anticoagulant medications	↑	↓	↑
Patient is very concerned about invasiveness or risks of lumbar puncture	↑	↓	↑
Patient has risk factors for a difficult lumbar puncture such as scoliosis, prior lumbar back surgery, or severe lumbar adiposity	↑	↓	↑
Patient's differential diagnosis includes non‐AD conditions that can be evaluated for with CSF tests	↓	↑	↓
Patient is a candidate for AD‐specific treatments and insurance requires CSF or amyloid PET for biomarker confirmation	↑	↑	↓
Patient can only access lower accuracy or poorly validated AD blood tests	↑	↑	↓
Patient has chronic kidney disease, liver cirrhosis, or prior myocardial infarction or stroke	↑	↑	↓

Abbreviations: AD, Alzheimer's disease; CSF, cerebrospinal fluid; PET, positron emission tomography.

## 5 INTERPRETATION OF AD BIOMARKER RESULTS

As biomarkers are used more broadly to determine whether individuals have AD pathology, the accuracy and validation of these tests must be scrutinized because of the major implications of the test results. Positive AD biomarkers often lead to a diagnosis of mild cognitive impairment or dementia due to AD, which are life‐altering conditions with profound implications for patients and their loved ones. Furthermore, decisions about whether to initiate expensive and burdensome treatments now depend on the results of biomarker tests.^19^ Therefore, these diagnoses and decisions require high accuracy tests that have been appropriately validated. Even screening tests must have relatively high levels of accuracy, as false negative results may delay care and false positive results may cause anxiety and increase the burden on specialty centers to perform follow‐up testing. Before ordering an AD biomarker test, clinicians must be aware of the accuracy and validation of the specific test they are using.

Even when clinicians use tests that accurately classify AD pathology in > 90% of patients, the certainty of positive and negative test results depends on factors specific to each individual patient. The pre‐test probability of AD pathology should significantly impact a clinician's confidence in a positive or negative result (Table 2). For example, a 75‐year‐old patient with a typical AD dementia syndrome has a high pre‐test probability of AD pathology and a positive test result is probably a true positive, whereas a negative result has a significant likelihood of being false negative. Patients that are thought to have a low pre‐test probability of AD typically do not undergo biomarker tests. However, in a patient with a low pre‐test probability of AD pathology (e.g., a 60‐year‐old patient with subjective cognitive decline), a negative test result is probably a true negative, whereas a positive result has a significant likelihood of being false positive. Therefore, clinicians must adhere to the adage to “treat the patient and not the test,” and consider the possibility of inaccurate test results when the clinical course is incongruent with the test result. Additional testing may be appropriate when confidence in the initial test result is low.

**TABLE 2:** Effect of pre‐test probability on the percentage of true positive/negative and false positive/negative results.

Patient	Pre‐test probability of amyloid pathology	Positive biomarker result	Negative biomarker result
75‐year‐old patient with a typical AD dementia syndrome	85%	98% of patients have a true positive result (have amyloid pathology)	61% of patients have a true negative result (do not have amyloid pathology)
		2% of patients have a false positive result (do not have amyloid pathology)	39% of patients have a false negative result (have amyloid pathology)
60‐year‐old patient with subjective cognitive decline	20%	69% of patients have a true positive result (have amyloid pathology)	97% of patients have a true negative result (do not have amyloid pathology)
		31% of patients have a false positive result (do not have amyloid pathology)	3% of patients have a false negative result (have amyloid pathology)

*Note*: The frequency of amyloid positivity for the two hypothetical patients is derived from data on the prevalence of amyloid positivity.^17^ The AD biomarker test is assumed to have 90% sensitivity and specificity for amyloid pathology.

Abbreviation: AD, Alzheimer's disease.

Different biomarker tests provide different types of results that affect clinical care. For example, amyloid PET scans are typically visually read as either positive or negative and a quantitative value is usually not provided. This output is simple and easy to understand, but patients and providers often would like a more detailed result that contextualizes the degree of biomarker abnormality. All clinically available CSF tests provide continuous values for biomarker measures, which allows clinicians to determine whether a patient has a clear positive, clear negative, or borderline result. One CSF test uses two cutoffs and categorizes individuals as positive, likely positive, and negative. The use of two cutoffs is helpful in identifying individuals with borderline levels of AD pathology who may drive discordance of biomarker results.^34^ However, it is unclear how clinicians should manage patients in this intermediate group; possibilities include performing a different test or re‐testing later. For patients who may be candidates for AD‐specific treatments, it may be appropriate to test patients with intermediate biomarker values with multiple testing modalities, because the result may have a major impact on their care. Overall, the use of two cutoffs and/or continuous measures may be helpful in personalizing care, but these approaches are likely to increase the complexity of the diagnostic process.

To interpret biomarker tests appropriately, clinicians must understand and use the measures that best correspond with AD pathology. For example, the Roche Elecsys CSF test provides measurements of Aβ42, p‐tau181, and t‐tau with reference ranges for these individual analytes. However, the measures that best classify amyloid status, and which are FDA‐approved for identification of AD pathology, are the ratios of p‐tau181 or t‐tau to Aβ42 (p‐tau181/Aβ42 or t‐tau/Aβ42). The clinical testing service typically provides guidance and reference ranges that instruct clinicians to use CSF p‐tau181/Aβ42 or t‐tau/Aβ42 to determine amyloid status. However, some providers may erroneously use CSF Aβ42 alone to determine amyloid status, even though this measure has a much lower association with amyloid pathology. There is even greater confusion about how to interpret some AD blood tests that provide a panel of results (e.g., Aβ42/Aβ40, p‐tau181, and neurofilament light [NfL]). In most cases, p‐tau181 or p‐tau217 more accurately classifies amyloid status than Aβ42/Aβ40.^35^, ^41^ Additionally, NfL is a non‐specific biomarker that is strongly correlated with age and may be negative in younger patients with AD pathology, and may be positive in older patients without AD pathology or with non‐AD conditions.^42^ Overall, proper interpretation of biomarker results requires careful review of test guidance and knowledge of the meaning of different results.

Clinicians must also understand that test results may be influenced by individual level factors such as race, ethnicity, and medical conditions. Validation studies of amyloid PET, tau PET, and CSF tests have primarily included healthy, well‐educated, non‐Hispanic White research participants. However, some studies have found that biomarker levels may vary by race and ethnicity,^43^, ^44^, ^45^ although other studies have not found racial or ethnic differences.^46^ There are also data demonstrating that some AD blood tests are affected by medical conditions^39^ and at least one medication.^47^ Given these issues, better understanding of biomarker performance in diverse populations is greatly needed and studies are currently underway. In the meantime, clinicians must exercise caution when using biomarker results in patients with demographic characteristics or medical conditions that have not been well represented in biomarker validation studies.

## 6 RETURNING AD BIOMARKER TEST RESULTS

Disclosure of AD biomarker results to patients is different from disclosure of other test results due to the sometimes poor prognosis and stigma associated with having a neurodegenerative disease. Therefore, whenever possible, we prefer face‐to‐face disclosure of biomarker results in a private setting with the presence of as many loved ones present as the patient wishes, although this is often not logistically possible. Based on our clinical experience, we recommend the following steps in disclosure of clinical AD biomarker results (Box 1). We generally open the disclosure process with a discussion of why the test was ordered, for example, “We performed this test to determine if Alzheimer's disease was likely to be the cause of your memory problems.” We then explicitly ask if the patient wants to learn the results now, obtaining consent to disclose. Once we have permission, we succinctly state the results, for example, “The test was positive for Alzheimer's disease,” and pause to give the patient a moment to process the result. We then describe our confidence in the accuracy of the test result, based on an understanding of the test's diagnostic performance and any added sources of uncertainty, such as if the patient is a member of a group or has clinical characteristics not well represented in studies validating the test. We then describe the impact of the result on the patient's diagnosis, for example, “Based on these results, I have a high degree of certainty that Alzheimer's disease is causing your memory problems.” This generally begins a discussion on prognosis, treatment options, or next steps if a diagnosis was not reached. Throughout the discussion, we frequently pause to create space for questions, and query the patient to ensure they understand the information, repeating key points in different ways if needed. Finally, we inform the patient of how additional questions can be answered after the disclosure session and provide options for follow‐up.

**BOX 1: Steps in returning AD biomarker test results**

1. Describe why the test was ordered.
2. Ask if the patient wants to learn the results now.
3. Provide a simple description of the results.
4. Describe the certainty of the results.
5. Connect the results to the clinical diagnosis.
6. Discuss prognosis, treatment options, and next steps.
7. Provide options for follow‐up.

**BOX 2: Potential uses of AD biomarkers related to AD‐specific treatments**

1. Confirmation that patients who may be candidates for AD‐specific treatments have AD pathology.
2. Longitudinal monitoring of biomarkers of AD pathology to determine whether treatments have reached their desired goal (e.g., amyloid clearance) and to guide decisions on whether to continue or stop treatments.
3. Monitoring biomarkers of neurodegeneration to reassure patients and clinicians that treatments are reducing neuronal damage.
4. Predicting and monitoring for complications of AD‐specific treatments, such as amyloid related imaging abnormalities.

## 7 FUTURE OF AD BIOMARKERS

As treatments specifically targeting AD pathology become more available, AD biomarker testing will become an increasingly important part of the routine clinical evaluation of cognitive impairment. Given the extremely limited number of dementia specialists and long wait times for an initial visit, it is likely that a timely diagnosis of early symptomatic AD will require biomarker testing to be performed by non‐dementia specialists such as general neurologists, geriatricians, and primary care providers. However, training non‐specialists to use AD biomarker tests is likely to be challenging, especially as the lay public and many clinicians often do not understand the basics of dementia. Many individuals conflate dementia and AD, and do not appreciate that dementia is an umbrella term that describes cognitive decline sufficiently severe to impair function in everyday activities, whereas AD is a specific brain disease characterized by amyloid plaques and tau tangles and is the most common cause of dementia. This understanding is essential to the appropriate use of AD biomarkers, which are used clinically to determine whether AD pathology is present and potentially causing cognitive impairment in a symptomatic patient. Educating clinicians and the public about dementia, AD, and biomarkers will be essential to enabling an earlier and more accurate diagnosis of AD dementia.

We expect the modalities used for biomarker testing will continue to shift over the next several years. CSF biomarkers have been the dominant modality of biomarker testing in most dementia specialty clinics because high accuracy tests were available and CSF testing has been reimbursed by insurance.^13^ However, the burden of CSF testing on providers is high,^24^ and many patients prefer brain imaging or blood tests. Now that Medicare has approved reimbursement for amyloid PET, an increasing number of patients are likely to undergo these scans. However, the acceptability, accessibility, and scalability of AD blood tests cannot be matched by other modalities, and AD blood tests are poised to become the dominant modality for clinical AD biomarker testing within the next 2 to 3 years. As multiple AD blood tests become clinically available and demonstrate high accuracy, clinicians will be more likely to order them and insurers will be more likely to pay for them, increasing their use. Due to their high acceptability and accessibility, we expect AD blood tests to decrease disparities in AD biomarker testing associated with race, ethnicity, socioeconomic factors, and location (rural vs. urban),^48^ and enable greater access to biomarker testing globally.

As AD‐specific treatments continue to advance, biomarkers will become integrated into all aspects of clinical dementia diagnosis and treatment (Box 2). In several years, treatments may be shown to slow or prevent symptom onset in cognitively unimpaired individuals with AD pathology;^21^, ^22^ if this occurs, screening cognitively unimpaired older individuals for AD pathology may be indicated. In the meantime, we expect the further development of CSF and plasma biomarkers that are strongly associated with cognitive impairment,^30^ which will help clinicians to better understand whether AD pathology is likely to be the cause of dementia. We expect that additional biomarkers of non‐AD dementias will be developed, which will improve our understanding of multiple etiologies of dementia as well as the effects of co‐pathologies on AD dementia.^49^, ^50^ Longitudinal evaluation of biomarkers of AD pathology could be used to determine whether treatments have reached their desired goal (e.g., amyloid clearance) and to guide decisions on whether to continue or stop treatments. Especially because current AD‐specific treatments are not expected to improve cognitive impairment, monitoring biomarkers of neurodegeneration could reassure patients and clinicians that treatments are reducing neuronal damage and could potentially enhance compliance with treatment.^11^ Further, there is a critical need for blood tests that could be used to predict and monitor for complications of AD‐specific treatments, such as amyloid related imaging abnormalities. Biomarkers have enabled the development of the first clinically available AD‐specific treatments, and biomarkers will increasingly become essential tools that will guide diagnosis, prognosis, and treatment decisions for patients with cognitive impairment.

## CONFLICT OF INTEREST STATEMENT

L. VandeVrede serves as a site principal investigator for Biogen‐sponsored clinical trials. S. Schindler reports serving on advisory boards for Eisai and receiving honoraria for lectures on biomarker testing. Washington University has an interest in C2N Diagnostics. S. Schindler has not received any compensation from C2N Diagnostics or any other diagnostics companies. Author disclosures are available in the supporting information.

## Supporting information

Supporting Information
