# Supplementary material for: Clinical use of biomarkers in the era of Alzheimer's disease treatments
Source: Alzheimers Dement. 2024 Dec 30;21(1):e14201. doi: 10.1002/alz.14201 (PMC11775455; doi:10.1002/alz.14201)
Supplement: Supplementary file 1 — Supporting Information [file ALZ-21-e14201-s001.pdf]

ICMJE DISCLOSURE FORM

**Date:**	5/24/2024
**Your Name:**	Suzanne E. Schindler
**Manuscript Title:**	Clinical use of biomarkers in the era of Alzheimer disease treatments
**Manuscript Number (if known):**	ADJ-D-24-00539

In the interest of transparency, we ask you to disclose all relationships/activities/interests listed below that are related to the content of your manuscript. "Related" means any relation with for-profit or not-for-profit third parties whose interests may be affected by the content of the manuscript. Disclosure represents a commitment to transparency and does not necessarily indicate a bias. If you are in doubt about whether to list a relationship/activity/interest, it is preferable that you do so.

The author's relationships/activities/interests should be defined broadly. For example, if your manuscript pertains to the epidemiology of hypertension, you should declare all relationships with manufacturers of antihypertensive medication, even if that medication is not mentioned in the manuscript.

In item #1 below, report all support for the work reported in this manuscript without time limit. For all other items, the time frame for disclosure is the past 36 months.

		**Name all entities with whom you have this relationship or indicate none (add rows as needed)**	**Specifications/Comments (e.g., if payments were made to you or to your institution)**
**Time frame: Since the initial planning of the work**			
1	All support for the present manuscript (e.g., funding, provision of study materials, medical writing, article processing charges, etc.)<br>**No time limit for this item.**	☒ **None**	
		National Institute on Aging grant R01AG070941 (SE Schindler)	
			Click the tab key to add additional rows.
**Time frame: past 36 months**			
2	Grants or contracts from any entity (if not indicated in item #1 above).	☐ **None**	
		Barnes-Jewish Hospital Foundation	
3	Royalties or licenses	☒ **None**	

		Name all entities with whom you have this relationship or indicate none (add rows as needed)	Specifications/Comments (e.g., if payments were made to you or to your institution)
**4**	Consulting fees	☐ **None**	
		Eisai	Advisory Boards on Blood-based biomarkers and biomarker education for providers, consulting on biomarker education
**5**	Payment or honoraria for lectures, presentations, speakers bureaus, manuscript writing or educational events	☐ **None**	
		University of Wisconsin, St. Luke's Hospital, Houston Methodist Medical Center, Weill Cornell, University of Massachusetts, Zucker School of Medicine, Medscape, (ATRI)/University of Southern California	Personal Honoraria for presenting lectures
		University of Washington	Personal Honoraria for serving on the Alzheimer Disease Center Clinical Task Force
		University of Indiana	Personal Honoraria for serving on the National Centralized Repository for Alzheimer's Disease biospecimen review committee
**6**	Payment for expert testimony	☒ **None**	
**7**	Support for attending meetings and/or travel	☐ **None**	
		National Institute on Aging grant R01AG070941 (SE Schindler)	Travel support is included in NIH grant
		Alzheimer's Association	Travel support for 2023 AAIC and 2023 Research Roundtable
		US Against Alzheimer's	Travel support for Lausanne X
**8**	Patents planned, issued or pending	☒ **None**	
**9**	Participation on a Data Safety Monitoring Board or Advisory Board	☒ **None**	
		World Health Organization	Participating in a committee advising the WHO on preferred product characteristics for fluid biomarkers of Alzheimer disease.
		University of Washington	Served on the Alzheimer Disease Center Clinical Task Force that is revising the data collection set used by all ADRCs; attended meetings every 2 weeks and did additional research and writing.

		Name all entities with whom you have this relationship or indicate none (add rows as needed)	Specifications/Comments (e.g., if payments were made to you or to your institution)
		University of Indiana	Reviewing sample requests for the National Centralized Repository for Alzheimer's Disease biospecimen review committee.
		University of Michigan	Member of the External Advisory Committee reviewing the Michigan ADRC and providing recommendations.
**10**	Leadership or fiduciary role in other board, society, committee or advocacy group, paid or unpaid	☐ **None**	
		Greater Missouri Chapter of the Alzheimer's Association	Board member working to support local efforts to raise funds for the Alzheimer's Association and advise them on research and support.
		Global CEO initiative workgroup on Blood-Based Biomarkers	Co-leader of workgroup tasked with developing performance standards for blood-based biomarkers; attended weekly meetings for ~6 months and worked on writing paper.
		Advisory Group on Risk Evaluation Education for Dementia	Participated in monthly calls discussing the ethical and legal implications of research on dementia that could allow for prediction of individual risk.
		Foundation for the National Institutes of Health Biomarkers Consortium	Project team member participating in planning head-to-head studies of blood-based biomarker assays.
**11**	Stock or stock options	☒ **None**	
**12**	Receipt of equipment, materials, drugs, medical writing, gifts or other services	☐ **None**	
		C2N Diagnostics	Plasma Ab42/Ab40 data was provided to Washington University by C2N Diagnostics at no cost. No payments/research funding was provided by C2N Diagnostics. No gifts/financial incentives of any kind have been provided to Dr. Schindler by C2N Diagnostics.
**13**	Other financial or non-financial interests	☒ **None**	

Please place an "X" next to the following statement to indicate your agreement:

☒ I certify that I have answered every question and have not altered the wording of any of the questions on this form.

ICMJE DISCLOSURE FORM

**Date:**	5/22/2024
**Your Name:**	Lawren VandeVrede
**Manuscript Title:**	Clinical use of biomarkers in the era of Alzheimer disease treatments
**Manuscript Number (if known):**	ADJ-D-24-00539

In the interest of transparency, we ask you to disclose all relationships/activities/interests listed below that are related to the content of your manuscript. "Related" means any relation with for-profit or not-for-profit third parties whose interests may be affected by the content of the manuscript. Disclosure represents a commitment to transparency and does not necessarily indicate a bias. If you are in doubt about whether to list a relationship/activity/interest, it is preferable that you do so.

The author's relationships/activities/interests should be defined broadly. For example, if your manuscript pertains to the epidemiology of hypertension, you should declare all relationships with manufacturers of antihypertensive medication, even if that medication is not mentioned in the manuscript.

In item #1 below, report all support for the work reported in this manuscript without time limit. For all other items, the time frame for disclosure is the past 36 months.

		**Name all entities with whom you have this relationship or indicate none (add rows as needed)**	**Specifications/Comments (e.g., if payments were made to you or to your institution)**
**Time frame: Since the initial planning of the work**			
1	All support for the present manuscript (e.g., funding, provision of study materials, medical writing, article processing charges, etc.) **No time limit for this item.**	☐ **None**	
		NIH K23AG073514	
		Alzheimer's Association	
		Shenandoah Foundation	
**Time frame: past 36 months**			
2	Grants or contracts from any entity (if not indicated in item #1 above).	☒ **None**	
3	Royalties or licenses	☒ **None**	

		Name all entities with whom you have this relationship or indicate none (add rows as needed)	Specifications/Comments (e.g., if payments were made to you or to your institution)
**4**	Consulting fees	☒ **None**	
**5**	Payment or honoraria for lectures, presentations, speakers bureaus, manuscript writing or educational events	☒ **None**	
**6**	Payment for expert testimony	☒ **None**	
**7**	Support for attending meetings and/or travel	☒ **None**	
**8**	Patents planned, issued or pending	☒ **None**	
**9**	Participation on a Data Safety Monitoring Board or Advisory Board	☒ **None**	
**10**	Leadership or fiduciary role in other board, society, committee or advocacy group, paid or unpaid	☒ **None**	

		Name all entities with whom you have this relationship or indicate none (add rows as needed)	Specifications/Comments (e.g., if payments were made to you or to your institution)
**11**	Stock or stock options	☒ **None**	
**12**	Receipt of equipment, materials, drugs, medical writing, gifts or other services	☒ **None**	
**13**	Other financial or non-financial interests	☒ **None**	

Please place an "X" next to the following statement to indicate your agreement:

☒ I certify that I have answered every question and have not altered the wording of any of the questions on this form.
